# The relationship between parental autonomy support, teacher autonomy support, peer support, and university students’ academic engagement: the mediating roles of basic psychological needs and autonomous motivation

**DOI:** 10.3389/fpsyg.2025.1503473

**Published:** 2025-04-28

**Authors:** Yan Li, Rosilawati Sueb, Khadijah Said Hashim

**Affiliations:** ^1^School of Language and Cultures, Youjiang Medical University for Nationalities, Baise, China; ^2^Faculty of Education, Universiti Teknologi MARA, UiTM Puncak Alam Campus, Selangor, Malaysia

**Keywords:** ecological systems theory, self-determination theory, environmental factors, individual factors, academic engagement, PLS-SEM

## Abstract

**Objective:**

This study, based on Ecological Systems Theory and Self-Determination Theory, explores the relationships between parental autonomy support, teacher autonomy support, peer support, and university students’ academic engagement from a positive psychology perspective, as well as the mediating roles of basic psychological needs and autonomous motivation.

**Methods:**

A questionnaire survey was conducted with 416 university students from four universities in Guangxi, using the Academic Engagement Scale, Parental Autonomy Support Scale, Teacher Autonomy Support Scale, Peer Support Scale, Basic Psychological Needs Scale, and Learning Motivation Scale.

**Results:**

(1) Teacher autonomy support was significantly positively associated with university students’ academic engagement; peer support was significantly negatively associated with academic engagement; parental autonomy support was not significantly associated with academic engagement. (2) Basic psychological needs significantly mediated the relationships between parental autonomy support, teacher autonomy support, peer support, and academic engagement. (3) Autonomous motivation significantly mediated the relationships between parental autonomy support, teacher autonomy support, and academic engagement, while it was not significantly associated with the relationship between peer support and academic engagement. (4) Basic psychological needs and autonomous motivation played a chain-mediating role in the relationships between parental autonomy support, teacher autonomy support, peer support, and academic engagement.

**Conclusion:**

Teacher, parental, and peer support influence university students’ academic engagement through different pathways, with basic psychological needs and autonomous motivation serving as important “bridging” factors.

## 1 Introduction

In recent years, with the widespread promotion and popularization of positive psychology (Seligman and Csikszentmihalyi, 2000), educators have increasingly recognized the positive impact of psychological strengths on individuals. There is a growing effort to explore students’ potential, strengths, and positive psychological states from the perspective of positive psychology, helping students develop through their internal resources. Academic engagement is a crucial psychological variable in positive psychology and has been a long-standing topic of research. Academic engagement refers to an individual’s fulfilling, stable, and sustained positive psychological state related to learning, which includes the dimensions of vigor, dedication, and absorption (Schaufeli et al., 2002).

Studies have shown that academic engagement significantly positively predicts various positive behaviors and psychological outcomes, such as academic achievement (Chen et al., 2023), school adaptation (Benito-Gomez et al., 2022), and well-being (Luruli et al., 2020). Similarly, academic engagement plays an important role in alleviating and moderating negative behaviors and psychological states, such as burnout (Abreu Alves et al., 2022), dropout intention (Virtanen et al., 2014), school truancy, and academic procrastination (Wei, 2023). Moreover, these positive behaviors, outcomes, or states can effectively promote students’ academic engagement forming a positive feedback loop.

Therefore, academic engagement, as an important reflection of the positive psychological aspect of students’ learning, can indicate students’ positive and healthy psychological states. Academic engagement is not only a core indicator for assessing educational quality and students’ development (Kuh, 2001; Trowler, 2010), but also a key predictor of academic success (Kahu and Nelson, 2018). With the transition of higher education in China from mass education to universal education, universities are gradually shifting their focus from the expansion of “quantity” to the enhancement of “quality” (Wang Z., 2023). In this educational context, paying attention to university students’ academic engagement has significant meaning and value.

In recent years, researchers have explored the influencing factors of academic engagement from multiple dimensions, mainly including environmental and individual factors. Based on Ecological Systems Theory, individual development is influenced by the joint effects of multi-level environmental factors. Previous studies have focused on the impact of micro-system factors (such as parents, teachers, and peers) on academic engagement (Chen et al., 2025b; Espinoza-Gutiérrez et al., 2024; López-García et al., 2022; Vargas-Madriz et al., 2024; Yang et al., 2023; Yang and Xiang, 2025). At the same time, some literature based on Self-Determination Theory emphasizes the key role of individual factors (basic psychological needs, autonomous motivation) in self-integration and positive development (Bai and Gu, 2024; Chen et al., 2025a; Du W. et al., 2023; Zhang and Qian, 2022). However, there are still some gaps: (1) Few studies have integrated the three important and multi-level environmental factors—parents, teachers, and peers—into the same research model to systematically explore their relationship with academic engagement. Most studies focus on the relationship between teachers or parents and academic engagement, while fewer explore the relationship between peers and academic engagement; (2) In the context of Chinese education, there is very limited research on the relationship between parents, teachers, peers, and academic engagement among university students, with most studies focusing on primary, middle, and high school students in the basic education stage; (3) There is little research that comprehensively and systematically combines environmental and individual factors to explore the influencing factors of academic engagement and the mechanisms between them.

Based on these research gaps, this study aims to integrate Ecological Systems Theory and Self-Determination Theory to explore the relationships between parental autonomy support, teacher autonomy support, peer support, and academic engagement among Chinese university students, as well as the independent and chain mediating roles of basic psychological needs and autonomous motivation. Through empirical research, this study will investigate how environmental support from different sources can enhance students’ academic engagement by satisfying basic psychological needs and stimulating autonomous motivation. This research not only contributes to a deeper understanding of the mechanisms behind academic engagement among university students, but also provides theoretical support and practical guidance for improving educational quality, student development, and home-school-peer collaborative intervention programs in higher education institutions.

## 2 Literature review and hypothesis development

### 2.1 Parental autonomy support, teacher autonomy support, peer support, and academic engagement

Ecological Systems Theory (Bronfenbrenner, 1979) posits that individual development occurs within a multi-level environmental system. These environmental factors range from the innermost micro-systems (such as family, school, and peers) to the outermost macro-systems (such as culture and society). According to Ecological Systems Theory, the most intrinsic environmental factors (such as parents, teachers, and peers) tend to have the most direct and strongest impact on individuals.

#### 2.1.1 Parental autonomy support and academic engagement

Parental autonomy support refers to parents’ ability to understand and respect their children’s unique internal feelings and needs, encouraging them to become active participants in their own behavior, thus experiencing a sense of control and belonging (Soenens et al., 2007). Specifically, parental autonomy support includes recognizing the child’s emotional experiences, providing reasonable explanations for rules and expectations, and offering opportunities for choice and autonomy (Mageau et al., 2015).

As a core element of the child’s developmental environment, parents’ behaviors have a profound impact on their children’s physical and mental development. Numerous studies have shown that parental autonomy support plays a positive role in children’s growth. Zhao et al. (2024) found through a longitudinal study that parental autonomy support helps cultivate children’s core self-evaluations, thereby enhancing their career adaptability and professional development potential. Moreover, the effects of parental autonomy support are not short-lived; they continuously and positively shape children’s long-term career development. Another longitudinal study indicated that parental autonomy support is an important protective factor in alleviating anxiety and depression symptoms (Dong et al., 2024). Parental autonomy support helps adolescents navigate adolescence healthily (Tunca, 2024). Martínez-López et al. (2024) found that family support is significantly positively correlated with students’ metacognitive knowledge, metacognition, cognitive learning strategies, and mastery-oriented self-talk. Perceived social support is an important resource for coping with academic challenges and helps students improve academic performance. In contrast, parental controlling support often has a negative impact on children’s learning, leading to significant declines in academic performance (Park et al., 2023).

Research has shown that parental autonomy support has a positive predictive effect on children’s academic engagement. Chen et al. (2025b) found in a study with Chinese high school students that parental autonomy support significantly positively predicted academic engagement. Parental autonomy support helps children set long-term learning goals effectively, and more importantly, children internalize external norms and rules into self-driven behavioral guidelines (Brauer, 2017), thereby enhancing academic engagement. Jiang et al. (2022) also found that parental autonomy support significantly positively predicted the academic engagement of vocational school students. For vocational students with relatively weak academic performance, receiving support, understanding, encouragement, and recognition from parents effectively alleviates the negative impact of external environments and helps them reshape their self-worth, thus maintaining academic motivation. Sağkal and Sönmez (2022) found that, in the case of mathematics, parental autonomy support had a direct and significant effect on middle school students’ mathematical engagement. Bas and Xu (2024) also pointed out that parental involvement can significantly predict students’ completion of assignments. Thus, parental autonomy support significantly predicts students’ academic engagement.

#### 2.1.2 Teacher autonomy support and academic engagement

Teacher autonomy support refers to the degree to which students perceive their teachers as instilling confidence in their abilities and making them feel understood, listened to, and accepted (Williams and Deci, 1996). It is a teaching approach that encourages students to think independently and solve problems, emphasizing the provision of necessary information and opportunities for choice, understanding students’ emotions, and avoiding the imposition of external control and pressure (Simon and Salanga, 2021).

As one of the most frequently encountered elements in students’ daily micro-systems, teachers play an extremely important and direct role in students’ learning activities. In the context of school education, teachers have an irreplaceable role in student development. Guoxia and Yang (2024) found through meta-analysis that teachers are the primary guides in educational activities, and teacher autonomy support is significantly positively correlated with students’ satisfaction of basic psychological needs, academic motivation, academic engagement, and academic achievement. When students perceive high levels of teacher closeness and autonomy support in the classroom, their use of deep learning strategies is also significantly enhanced Schweder and Raufelder (2022). When teachers pay attention to students’ interests, provide personalized feedback, and offer more opportunities for learning choices (Schweder and Raufelder, 2022), they not only enhance students’ positive emotional experiences but also indirectly promote deep learning by boosting academic self-efficacy.

Sueb et al. (2020) noted that students’ willingness to learn is greatly influenced by how effectively teachers manage students and the classroom. Excellent teachers use their years of accumulated experience to effectively manage and inspire students, helping them engage in learning. López-García et al. (2022) stated that even in online education, teacher autonomy-supportive teaching styles (such as offering choices, cooperative learning, and personalized feedback) are significantly positively predictive of academic engagement. Parker et al. (2021) also found that perceived teacher autonomy support has a significant positive predictive effect on students’ classroom participation. When teachers provide autonomy, competence, and emotional support, positive teacher-student interactions stimulate stronger student engagement (Guo et al., 2023). Tao et al. (2022a) concluded through meta-analysis that teacher autonomy support has the greatest impact on the academic performance of high school students. Therefore, teacher autonomy support has a huge impact on students’ academic engagement (Pan and Yao, 2023).

Thus, teacher autonomy support is closely related to students’ academic engagement and plays an important role in student development.

#### 2.1.3 Peer support and academic engagement

Peer support refers to the emotional care, companionship, and academic and life assistance individuals receive from their peers during interactions with those of the same age (Wentzel et al., 2010). As an important social support resource within the micro-system, peer support plays a significant role in various aspects of students’ growth. Peer support can alleviate the negative effects associated with externalizing behavioral problems in rural left-behind children in China (Zhang et al., 2021). Peer support also promotes students in forming more appropriate academic cognitions and more positive academic emotions (Ulmanen et al., 2024), enhances emotional regulation abilities, and thus improves academic performance (Xie and Guo, 2023), as well as increases students’ psychological well-being (Hoferichter et al., 2021).

Research has shown that peer support is significantly positively associated with academic engagement (Yang and Xiang, 2025). The more peer support university students receive, the higher their level of academic engagement. Peer support is an important environmental factor associated with the positive development of university students. When university students face academic difficulties, receiving encouragement and supportive information from peers can enhance their academic engagement and success (Yang and Xiang, 2025). Particularly for students with self-regulation difficulties, the importance of peer support in learning is emphasized, as peer support helps improve skills such as planning, monitoring, and reflection (Räisänen et al., 2021). Peer support, as a learning resource, can motivate individuals to engage more actively in learning and development (Yang and Xiang, 2025). This demonstrates that peer support is an important positive environmental factor associated with academic engagement among university students.

In summary, the following hypotheses are proposed:

*H1*: Parental autonomy support, teacher autonomy support, and peer support are positively associated with academic engagement in university students.

*H1a*: Parental autonomy support is positively associated with academic engagement in university students.

*H1b*: Teacher autonomy support is positively associated with academic engagement in university students.

*H1c*: Peer support is positively associated with academic engagement in university students.

### 2.2 The mediating role of basic psychological needs and autonomous motivation

Basic psychological needs and autonomous motivation are core concepts in Self-Determination Theory (SDT). Self-Determination Theory was proposed by American psychologists Deci and Ryan (1985). The theory posits that every individual has an inherent potential for self-actualization and personal growth, along with a tendency to continuously integrate the self. However, this intrinsic growth potential does not automatically manifest; it requires external environmental support and nourishment to be realized (Ryan and Deci, 2017).

According to Self-Determination Theory, individuals have three innate, fundamental psychological needs: autonomy, competence, and relatedness (Deci and Ryan, 1985). Basic psychological needs function like “nutrients” for psychological growth, providing continuous energy for individual development, integration, and well-being. When the external environment provides autonomy support, these basic psychological needs can be satisfied, promoting positive development and self-actualization. Conversely, when the external environment lacks support or suppresses these needs, individual development will be hindered (Ryan and Deci, 2017; Vansteenkiste et al., 2020).

Self-Determination Theory asserts that the satisfaction of basic psychological needs is a crucial source for fostering autonomous motivation in individuals (Deci and Ryan, 2000; Vansteenkiste et al., 2020). When basic psychological needs are met, individuals are more likely to exhibit spontaneous, intrinsically driven behaviors (Ryan and Deci, 2020), which allows them to better realize their potential, demonstrate strengths, and uncover positive psychological qualities (Ryan and Deci, 2020).

Self-Determination Theory provides a solid theoretical foundation for understanding the mediating role of basic psychological needs and autonomous motivation between autonomy-supportive environments and individual development.

#### 2.2.1 Mediating role of basic psychological needs

Self-Determination Theory has demonstrated through numerous empirical studies that among all psychological needs, autonomy, competence, and relatedness are the three basic psychological need (Ryan and Deci, 2000). Autonomy refers to the ability of individuals to freely choose and control their own behaviors; competence refers to the sense of self-efficacy individuals feel when interacting with their environment; and relatedness refers to the desire to establish meaningful social connections with others (Ryan and Deci, 2017). Based on the organic dialectical theory, Self-Determination Theory posits that every individual has the potential for upward growth, but this potential requires external environmental support. In a supportive environment, individuals can satisfy their basic psychological needs, which makes it easier for them to demonstrate healthy psychological growth and realize their potential. However, in a constraining environment, when individuals’ basic psychological needs are suppressed, their development is hindered, and they may experience psychological stagnation or pathological phenomena (Ryan and Deci, 2017; Vansteenkiste et al., 2020).

Self-Determination Theory emphasizes the important role of the external environment in fulfilling basic psychological needs. The theory suggests that an environment providing autonomy support is conducive to meeting individuals’ basic psychological needs, thereby promoting positive development (Ryan and Deci, 2017). Du W. et al. (2023) suggests that good parent–child interactions help children explore their interests and develop their abilities freely, promoting positive development by meeting their basic psychological needs and enhancing their learning resilience, which enables them to engage in learning activities (Lan, 2023). Studies by Xu et al. (2024) and Xin (2022) mention that basic psychological needs play a crucial mediating role. When students perceive social support or teacher autonomy support, meeting their basic psychological needs can promote their engagement. A longitudinal study conducted over 2 years Yu et al. (2015), found that teacher autonomy support increased students’ basic psychological needs, reduced students’ excessive dependence on online games, and thereby improved their school participation.

However, if the social environment hinders the satisfaction of an individual’s basic psychological needs, it can have negative effects on the individual, such as seeking substitutes for needs, engaging in compensatory behaviors, etc. (Ryan and Deci, 2017; Tabiś and Poprawa, 2023; Yu et al., 2024), all of which are detrimental to the individual’s healthy and positive development.

Therefore, an autonomy-supportive environment is conducive to the satisfaction of individuals’ basic psychological needs, which in turn promotes their development in a favorable direction and alleviates and moderates negative behavioral consequences.

Thus, the following hypothesis is proposed:

*H2*: Basic psychological needs mediate the relationship between parental autonomy support, teacher autonomy support, peer support, and academic engagement.

*H2a*: Basic psychological needs mediate the relationship between parental autonomy support and academic engagement.

*H2b*: Basic psychological needs mediate the relationship between teacher autonomy support and academic engagement.

*H2c*: Basic psychological needs mediate the relationship between peer support and academic engagement.

#### 2.2.2 Mediating role of autonomous motivation

Self-Determination Theory posits that autonomous motivation refers to the behavior that is considered autonomous when an individual feels voluntary, fully identifies with, and willingly engages in a certain activity (Ryan and Deci, 2017). In other words, individuals make decisions and choices based on their own desires and thoughts, which is considered a positive psychological quality (Soenens and Vansteenkiste, 2005). In educational learning contexts, autonomous motivation is a crucial determinant for promoting meaningful learning and deep learning (Adam et al., 2023). Individuals who exhibit autonomous motivation are more likely to recognize learning as something meaningful, valued by parents, teachers, and society, and integrate this view into their self-concept, thereby becoming more engaged in learning. The higher the level of an individual’s self-determined motivation, the more they are able to maintain an autonomous and positive state when facing learning tasks, leading to better adaptation to learning.

Studies have found that autonomous motivation plays a significant mediating role in the relationship between an autonomy-supportive learning environment and academic engagement. Yan et al. (2025) found that the teacher autonomy support style significantly influenced the persistence of vocational students in sports through autonomous motivation. Yang and Du (2023) found that autonomous motivation mediated the relationship between perceived teacher autonomy support and online learning engagement, whereas controlled motivation did not. This indicates that an autonomy-supportive learning environment helps foster autonomous motivation, leading to higher self-drive, more sustained academic engagement, and a higher quality learning experience (Ryan and Deci, 2017).

Thus, autonomous motivation is considered a mediating variable between environmental factors and individual development. In summary, the following hypothesis is proposed:

*H3*: Autonomous motivation mediates the relationship between parental autonomy support, teacher autonomy support, peer support, and academic engagement.

*H3a*: Autonomous motivation mediates the relationship between parental autonomy support and academic engagement.

*H3b*: Autonomous motivation mediates the relationship between teacher autonomy support and academic engagement.

*H3c*: Autonomous motivation mediates the relationship between peer support and academic engagement.

#### 2.2.3 The chain mediating role of basic psychological needs and autonomous motivation

Self-Determination Theory posits that there is a close relationship between basic psychological needs and autonomous motivation, with the satisfaction of basic psychological needs being a key factor in the development of autonomous motivation (Ryan and Deci, 2017, 2020). Early experiments in Self-Determination Theory indicated that a supportive environment is conducive to the satisfaction of basic psychological needs, which in turn promotes intrinsic motivation (Deci and Ryan, 1985). An environment that supports the satisfaction of basic psychological needs not only helps develop intrinsic motivation but also promotes the internalization of extrinsic motivation.

In the field of education, previous studies have shown that basic psychological needs and autonomous motivation play a significant mediating role in the relationship between autonomy-supportive environments and individual development. Research by Siacor and Ng (2024) found that teacher autonomy support helps meet students’ psychological needs and ultimately stimulates students’ learning motivation and engagement. Zhang and Qian (2022) discovered that teacher autonomy support promotes students’ academic engagement through the chain mediating role of basic psychological needs and autonomous motivation.

In summary, the following hypothesis is proposed:

*H4*: Basic psychological needs and autonomous motivation play a chain mediating role in the relationship between parental autonomy support, teacher autonomy support, peer support, and academic engagement.

*H4a*: Basic psychological needs and autonomous motivation play a chain mediating role in the relationship between parental autonomy support and academic engagement.

*H4b*: Basic psychological needs and autonomous motivation play a chain mediating role in the relationship between teacher autonomy support and academic engagement.

*H4c*: Basic psychological needs and autonomous motivation play a chain mediating role in the relationship between peer support and academic engagement.

## 3 Methods

### 3.1 Sample and procedure

The Strengthening the Reporting of Observational Studies in Epidemiology (STROBE) Initiative (Sánchez-Martín et al., 2024; Von Elm et al., 2008) was used for the study description. Prior to data collection, approval was obtained from the Ethics Committee of Youjiang Medical University for Nationalities, with the ethics review number 2023043001. Before data collection, all participants voluntarily signed an informed consent form. To reduce response bias (Lazarsfeld et al., 1968; Mayer, 2021), the researcher explained the content and purpose of the study in a friendly tone when distributing the questionnaires. It was emphasized that the survey was anonymous, the data would only be used for scientific research, there were no right or wrong answers, and participants were to respond based on their actual situation. Participants voluntarily participated in the survey and were free to discontinue it at any time without explanation or any adverse consequences. To enhance participants’ motivation and the quality of the questionnaires, a gift was prepared for each participant.

All six questionnaires used in this study were developed by foreign scholars, with the original language being English. As the participants were Chinese university students, the questionnaires needed to be translated into Chinese to ensure that each participant could accurately understand the content and provide truthful responses (Lee et al., 1999). The study employed a back-translation method, strictly following the principles of back-translation to ensure the rigor and accuracy of the translation process (Brislin, 1970).

To ensure the reliability, validity, and operability of the scales, a pilot study was conducted before the formal survey. In the pilot study, a total of 52 questionnaires were distributed. Based on the participants’ feedback, necessary adjustments and optimizations were made, and the final version of the questionnaire was determined.

Subsequently, from April to May 2024, formal surveys were conducted at four universities in Guangxi. A total of 437 paper-based questionnaires were distributed using cluster sampling across the four universities. The final number of valid questionnaires returned was 416, resulting in an effective response rate of 95.19%. Invalid questionnaires were excluded based on the following criteria: (1) straight-lining, where all items were answered with the same option (Krosnick, 1991); (2) excessive missing data, where more than 15% of the items were left blank consecutively (Little and Rubin, 2019); (3) failure to select the correct option for attention-check questions, which were inserted to test participants’ attention. If the specified option was not selected, it indicated inattentive answering and poor questionnaire quality (Meade and Craig, 2012; Oppenheimer et al., 2009).

### 3.2 Measures

#### 3.2.1 Demographic survey

Demographic information was collected through a self-developed questionnaire, including gender, age, grade, family location, and whether the participant is an only child.

#### 3.2.2 Academic engagement: Utrecht Work Engagement Scale-Student Version (UWES)

The Utrecht Work Engagement Scale-Student Version (UWES) was developed by Schaufeli et al. (2002). This study used the UWES-9 version (Schaufeli et al., 2006). The scale includes three dimensions: vigor, dedication, and absorption. The scale uses a Likert 6-point scoring system, ranging from 0 (Never) to 6 (Always). The study used the average score for calculation, with higher scores indicating higher levels of academic engagement.

#### 3.2.3 Parental autonomy support: Perceived Parental Autonomy Support Scale (P-PASS)

The Perceived Parental Autonomy Support Scale (P-PASS) was developed by Mageau et al. (2015). The scale includes two aspects: parental autonomy support and control strategies. This study selected the parental autonomy support section to measure the extent to which children perceive parental autonomy support. The parental autonomy support questionnaire consists of 12 items, covering three dimensions: Offering choice, Explaining reasons, and Accepting feelings. It uses a Likert 7-point scale, ranging from 1 (Do not agree at all) to 7 (Very strongly agree). This study used the average score for calculation, with higher scores indicating a higher level of perceived parental autonomy support.

#### 3.2.4 Teacher autonomy support: Learning Climate Questionnaire (LCQ)

The Learning Climate Questionnaire (LCQ) was developed by Williams and Deci (1996). This study used the 5-item version of the scale (LCQ-5) (Simon and Salanga, 2021), which assesses students’ perception of the level of autonomy support provided by teachers. The scale is unidimensional and uses a Likert-7 point scoring system, ranging from 1 (strongly disagree) to 7 (strongly agree). This study used the average score for calculation, with higher scores indicating higher levels of perceived teacher autonomy support.

#### 3.2.5 Peer support: Multidimensional Scale of Perceived Social Support (MSPSS)

The Multidimensional Scale of Perceived Social Support (MSPSS) was developed by Zimet et al. (1988). The scale includes support from family, friends, and other significant others. This study selected the peer support dimension to measure individuals’ perception of support from their peers. The peer support dimension consists of 4 items and uses a Likert-7 point scoring system, ranging from 1 (strongly disagree) to 7 (strongly agree). This study used the average score for calculation, with higher scores indicating higher levels of perceived peer support.

#### 3.2.6 Basic psychological needs: Basic Psychological Needs Scale (BPNS)

The Basic Psychological Needs Scale (BPNS) was developed by Gagné (2003). It consists of 21 items across three dimensions: Autonomy Need (7 items), Competence Need (6 items), and Relatedness Need (8 items). Ten items in the scale are reverse-scored. The scale uses a Likert-7 point scoring system, ranging from 1 (strongly disagree) to 7 (strongly agree). This study used the average score for calculation, with higher scores indicating higher levels of satisfaction of basic psychological needs.

#### 3.2.7 Autonomous motivation: Academic Motivation Scale (AMS)

The Academic Motivation Scale (AMS) was developed by Vallerand et al. (1992). This study used the 14-item version of the Academic Motivation Scale (AMS-14) (Kotera et al., 2023). The scale includes both autonomous motivation and controlled motivation. This study selected the autonomous motivation section to measure students’ level of autonomous motivation in learning. Autonomous motivation includes four dimensions: intrinsic motivation to know, intrinsic motivation toward accomplishment, intrinsic motivation to experience stimulation, and identified regulation. The scale uses a Likert-7 point scoring system, ranging from 1 (not at all true) to 7 (very true). This study used the average score for calculation, with higher scores indicating higher levels of autonomous motivation.

### 3.3 Data analysis

Data entry was performed using EpiData 3.1 software; data organization and descriptive statistical analysis were conducted using SPSS 26.0 software; and the reliability, validity, and research hypotheses of the scales were tested using Smart-PLS 4.0 software based on the Partial Least Squares Structural Equation Modeling (PLS-SEM) approach.

The use of PLS-SEM in this study is primarily based on the following considerations: First, the model in this study is relatively complex, involving multiple latent constructs, numerous measurement indicators, and complex path relationships (Chin, 2009; Hair et al., 2021). Secondly, this study is based on Ecological Systems Theory and Self-Determination Theory, making it a theoretical integrative exploratory study (Chin, 2009; Hair et al., 2021). Therefore, PLS-SEM, as a method suitable for complex models and exploratory research, is widely used in predictive modeling and path analysis research, which aligns well with the objectives of this study.

According to (Hair et al., 2014), the analysis process of PLS-SEM includes two stages: measurement model evaluation and structural model evaluation. The main goal of measurement model evaluation is to ensure that the latent variables have good reliability and validity. Since the model in this study is a second-order reflective construct model, following the recommendations of Bagozzi and Yi (1988) and Hair et al. (2017), it is necessary to assess the reliability and validity of both the first-order and second-order constructs to ensure the overall reliability and validity of the measurement model. The measurement model evaluation primarily includes four aspects: indicator reliability (outer loadings), internal consistency reliability (Cronbach’s *α* and composite reliability), convergent validity (Average Variance Extracted, AVE), and discriminant validity (Fornell-Larcker criterion and heterotrait-monotrait ratio, HTMT) (Hair et al., 2017; Hair et al., 2021).

After completing the measurement model evaluation, the structural model evaluation phase begins to test whether the path relationships between latent constructs are significant and to validate whether the research hypotheses are supported. Structural model evaluation primarily includes several aspects: multicollinearity test (VIF), path coefficient significance test (using 5,000 bootstrapping iterations), model explanatory power (R^2^), predictive relevance (Q^2^), effect size (f^2^), and model fit (such as SRMR, GOF) (Hair et al., 2017; Hair et al., 2021).

## 4 Results

### 4.1 Participants’ profile

The participants in this survey were aged between 18 and 25, with an average age of 20.96 ± 1.466 (M ± SD) years. Among the participants, 144 were male (34.6%) and 272 were female (65.4%). The sample included 86 first-year students (20.7%), 80 second-year students (19.2%), 117 third-year students (28.1%), and 133 fourth-year students (32.0%). In terms of geographic location, 240 were from rural areas (57.7%), 105 from suburban areas (25.2%), and 71 from urban areas (17.1%). There were 69 only children (16.6%) and 347 non-only children (83.4%). Detailed information is shown in Table 1.

**Table 1:** Demographics information of respondents.

Demographic	Groups	Frequency	Percentage (%)
Gender	Male	144	34.6
	Female	272	65.4
Age	18	17	4.1
	19	59	14.2
	20	84	20.2
	21	93	22.4
	22	101	24.3
	23	52	12.5
	24	7	1.7
	25	3	0.7
Academic year	First-year student	86	20.7
	Second-year student	80	19.2
	Third-year student	117	28.1
	Forth-year student	133	32.0
Type of residence	Rural	240	57.7
	Suburban	105	25.2
	Urban	71	17.1
Only child status	Yes	69	16.6
	No	347	83.4

### 4.2 Common method Bias

Since the data for this survey were all self-reported by the participants, there may be a risk of common method bias (Podsakoff et al., 2003). In this study, Harman’s single-factor test was used to perform an exploratory factor analysis on all items of the questionnaire (Harman, 1976). The analysis revealed that there were 13 factors with eigenvalues greater than 1, and the variance explained by the first factor was 25.26%, which is below the 40% critical value threshold. Therefore, it can be concluded that there is no common method bias in the data of this study.

### 4.3 Descriptive analysis and correlations

Table 2 shows the mean, standard deviation, and results of the correlation analysis for all variables. Pearson correlation analysis revealed significant positive correlations between parental autonomy support, teacher autonomy support, peer support, basic psychological needs, autonomous motivation, and academic engagement.

**Table 2:** Descriptive statistics of each variable and the results of correlation analysis.

Construct	M	SD	1	2	3	4	5	6
1. Parental autonomy support	5.22	1.08	1					
2. Teacher autonomy support	5.21	1.05	0.360**	1				
3. Peer support	5.72	1.12	0.363**	0.364**	1			
4. Basic psychological needs	4.79	0.80	0.390**	0.395**	0.494**	1		
5. Autonomous motivation	5.47	0.92	0.400**	0.564**	0.354**	0.444**	1	
6. Academic engagement	3.95	0.84	0.317**	0.457**	0.218**	0.395**	0.605**	1

**p < 0.01.

### 4.4 Assessment of measurement model

According to Hair et al. (2021), the assessment of a reflective measurement model is divided into four steps: (1) Assess the indicator reliability, (2) Assess the internal consistency reliability, (3) Assess the convergent validity, and (4) Assess the discriminant validity. The following sections will evaluate each step separately.

#### 4.4.1 Indicator reliability

The first step is to assess indicator reliability. Indicator reliability reflects the extent to which the variance of each indicator is explained by its construct, typically assessed using outer loadings (Hair et al., 2021). According to the recommendations of Hair et al. (2021), the evaluation criteria are as follows: (1) Outer loadings above 0.708 indicate good indicator reliability. (2) Outer loadings between 0.40 and 0.708 suggest further evaluation of the impact of removing the item on the internal consistency reliability and convergent validity of other indicators. If the removal significantly improves the composite reliability or AVE of the latent construct, it may be considered for removal. Additionally, when deciding whether to remove a measurement item, the impact on content validity should also be considered. If removing an item would result in certain key dimensions of the construct not being measured, the item should be retained, if necessary (Hair et al., 2021). (3) Outer loadings below 0.40 should be removed from the measurement model (Hair et al., 2021).

In this study, based on the above criteria, 8 items from the Basic Psychological Needs Scale were removed (CN3, CN5, AN2, AN4, AN7, RN3, RN4, RN6). These items had outer loading values between 0.40 and 0.708, and their removal significantly improved the convergent validity (AVE) of the constructs.

#### 4.4.2 Internal consistency reliability

The second step is to assess internal consistency reliability. Internal consistency reliability reflects the degree of correlation among the indicators of the same construct. It is typically assessed using Cronbach’s alpha and composite reliability as evaluation indicators. According to Hair et al. (2021), the evaluation criteria for Cronbach’s alpha and composite reliability are as follows: 0.60–0.70 is acceptable, 0.7–0.9 is satisfactory, and values above 0.95 indicate potential redundancy among the indicators.

As shown in Tables 3, 4, both the first-order constructs and the second-order constructs exhibit Cronbach’s alpha and composite reliability values above 0.7, and none exceed 0.95. Although the Cronbach’s alpha for CN1 and AN3 are 0.651 and 0.663, respectively, these values are still within the acceptable range (>0.6). The results indicate that both the first-order and second-order constructs in this study demonstrate good internal consistency reliability.

**Table 3:** Reliability and convergent validity of lower-order construct.

Item	Factor loading	Alpha	CR	AVE
Vigor		0.851	0.911	0.773
VI1	0.914			
VI2	0.917			
VI3	0.802			
Dedication		0.791	0.878	0.706
DE1	0.876			
DE2	0.853			
DE3	0.788			
Absorption		0.712	0.839	0.635
AB1	0.775			
AB2	0.840			
AB3	0.775			
Offering Choice		0.817	0.879	0.646
OC1	0.748			
OC2	0.767			
OC3	0.835			
OC4	0.860			
Explaining Reasons		0.893	0.926	0.758
EXR1	0.872			
EXR2	0.891			
EXR3	0.887			
EXR4	0.831			
Accepting Feelings		0.895	0.927	0.760
AF1	0.835			
AF2	0.875			
AF3	0.895			
AF4	0.881			
Teacher Autonomy Support		0.897	0.924	0.708
TS1	0.804			
TS2	0.862			
TS3	0.858			
TS4	0.862			
TS5	0.821			
Peer Support		0.900	0.930	0.769
PS1	0.881			
PS2	0.902			
PS3	0.885			
PS4	0.838			
Competence Need		0.695	0.812	0.521
CN1	0.651			
CN2	0.713			
CN4	0.753			
CN6	0.762			
Autonomy Need		0.697	0.815	0.525
AN1	0.712			
AN3	0.663			
AN5	0.766			
AN6	0.753			
Relatedness Need		0.799	0.862	0.556
RN1	0.749			
RN2	0.742			
RN5	0.718			
RN7	0.710			
RN8	0.804			
Intrinsic motivation to know		0.806	0.911	0.837
IMK1	0.915			
IMK2	0.915			
Intrinsic motivation to accomplishment		0.720	0.877	0.781
IMA1	0.883			
IMA1	0.884			
Intrinsic motivation to experience stimulation		0.803	0.910	0.836
IMS1	0.908			
IMS2	0.920			
Identified regulation		0.752	0.890	0.801
IDR1	0.885			
IDR2	0.905			

**Table 4:** Reliability and convergent validity of higher-order construct.

Construct	Factor loading	Alpha	CR	AVE
Academic engagement		0.872	0.922	0.797
VI	0.872			
DE	0.917			
AB	0.872			
Parental autonomy support		0.852	0.910	0.772
OC	0.879			
EXR	0.857			
AF	0.900			
Basic psychological needs		0.795	0.880	0.710
AN	0.889			
CN	0.763			
RN	0.870			
Autonomous motivation		0.801	0.872	0.633
IMK	0.901			
IMA	0.865			
IMS	0.649			
IDR	0.743			

#### 4.4.3 Convergent validity

The third step is to assess convergent validity. Convergent validity reflects the extent to which the measurement items of each construct converge and explain the variance of the construct’s indicators. It is typically assessed using Average Variance Extracted (AVE). According to Hair et al. (2021), an AVE value ≥ 0.50 indicates that the construct explains 50% or more of the variance in its measurement indicators, thus demonstrating good convergent validity.

In this study, as shown in Tables 3, 4, the AVE values for both first-order and second-order constructs are all greater than 0.50 (ranging from 0.521 to 0.837), indicating that all constructs exhibit good convergent validity.

#### 4.4.4 Discriminant validity

The fourth step is to assess discriminant validity. Discriminant validity reflects whether there is statistical distinction between the latent variables. Common methods for assessment include the Fornell-Larcker criterion (Fornell and Larcker, 1981) and the Heterotrait-Monotrait Ratio (HTMT) (Henseler et al., 2015). The evaluation criterion for the Fornell-Larcker criterion is that the AVE of each construct should be greater than the square correlation between that construct and the other constructs, indicating good discriminant validity. For HTMT, the evaluation criteria, according to Henseler et al. (2015) and Hair et al. (2021), are: HTMT value < 0.85 (strict standard), HTMT value < 0.9 (conservative standard), HTMT value < 1 (relaxed standard).

In this study, as shown in Tables 5, 6, both the first-order and second-order constructs have AVE values greater than the square correlations between constructs. Tables 7, 8 show that the HTMT values for most constructs, both first-order and second-order, are below 0.85. Although some constructs have higher correlations, they still meet the relaxed standard, with HTMT values less than 1 (Hair et al., 2021; Henseler et al., 2015). Based on the Fornell-Larcker criterion and the HTMT test results, the model in this study demonstrates acceptable discriminant validity.

**Table 5:** Fornell-Larcher criterion of the lower-order construct.

	AB	AF	AN	CN	DE	EXR	IDR	IMA	IMK	IMS	OC	PS	RN	TAS	VI
AB	**0.797**														
AF	0.263	**0.872**													
AN	0.32	0.461	**0.724**												
CN	0.294	0.273	0.528	**0.722**											
DE	0.707	0.237	0.329	0.332	**0.84**										
EXR	0.277	0.633	0.463	0.258	0.236	**0.87**									
IDR	0.41	0.302	0.311	0.243	0.384	0.31	**0.895**								
IMA	0.414	0.284	0.424	0.286	0.505	0.271	0.483	**0.884**							
IMK	0.489	0.349	0.446	0.342	0.54	0.346	0.592	0.735	**0.915**						
IMS	0.342	0.205	0.289	0.204	0.366	0.225	0.263	0.484	0.451	**0.914**					
OC	0.249	0.739	0.422	0.223	0.214	0.603	0.286	0.25	0.304	0.187	**0.804**				
PS	0.177	0.322	0.485	0.361	0.189	0.316	0.295	0.326	0.329	0.181	0.322	**0.877**			
RN	0.249	0.315	0.678	0.483	0.27	0.355	0.329	0.408	0.404	0.227	0.298	0.612	**0.745**		
TAS	0.412	0.349	0.392	0.279	0.408	0.314	0.441	0.477	0.527	0.343	0.287	0.371	0.413	**0.842**	
VI	0.638	0.248	0.376	0.421	0.74	0.279	0.386	0.437	0.527	0.338	0.222	0.234	0.251	0.417	**0.879**

The bold diagonal values refer to the square root of the AVE of each construct.

**Table 6:** Fornell-Larker Values of the Higher-Order Construct.

Construct	1	2	3	4	5	6
1. Parental autonomy support	**0.879**					
2. Teacher autonomy support	0.362	**0.842**				
3. Peer support	0.365	0.370	**0.877**			
4. Basic psychological needs	0.470	0.434	0.584	**0.842**		
5. Autonomous motivation	0.402	0.339	0.362	0.499	**0.796**	
6. Academic engagement	0.316	0.462	0.224	0.415	0.61	**0.893**

The bold diagonal values refer to the square root of the AVE of each construct.

**Table 7:** Discriminant validity of lower-order construct (HTMT).

	AB	AF	AN	CN	DE	EXR	IDR	IMA	IMK	IMS	OC	PS	RN	TAS	VI
AB															
AF	0.333														
AN	0.46	0.58													
CN	0.419	0.326	0.733												
DE	0.94	0.279	0.44	0.422											
EXR	0.348	0.707	0.589	0.314	0.278										
IDR	0.559	0.368	0.435	0.325	0.494	0.379									
IMA	0.576	0.354	0.602	0.381	0.668	0.34	0.657								
IMK	0.646	0.411	0.599	0.443	0.671	0.409	0.757	0.965							
IMS	0.453	0.241	0.388	0.257	0.459	0.267	0.338	0.636	0.559						
OC	0.331	0.86	0.555	0.28	0.26	0.70	0.36	0.324	0.373	0.233					
PS	0.22	0.36	0.604	0.433	0.219	0.352	0.363	0.405	0.386	0.211	0.374				
RN	0.332	0.374	0.901	0.617	0.338	0.422	0.424	0.541	0.506	0.288	0.368	0.723			
TAS	0.512	0.389	0.492	0.336	0.476	0.35	0.536	0.591	0.617	0.404	0.333	0.408	0.486		
VI	0.819	0.285	0.49	0.536	0.892	0.319	0.481	0.56	0.637	0.409	0.267	0.264	0.304	0.476	

**Table 8:** Discriminant validity of higher-order construct (HTMT).

Construct	1	2	3	4	5	6
1. Parental support						
2. Teacher support	0.41					
3. Peer support	0.416	0.408				
4. Basic psychological needs	0.560	0.506	0.681			
5. Autonomous motivation	0.481	0.665	0.420	0.614		
6. Academic engagement	0.365	0.519	0.252	0.505	0.726	

### 4.5 Assessment of structural model

After confirming that the reliability and validity of the measurement model meet the requirements, it is necessary to further assess the structural model to test the path relationships between the latent variables.

The first step is to check whether there are any potential multicollinearity issues between the predictor constructs and each endogenous construct in the structural model. If there is significant multicollinearity, it may lead to biased estimates of the path coefficients, affecting the explanatory power and stability of the structural model. Multicollinearity issues are typically assessed by checking the variance inflation factor (VIF) of the predictor variables (Hair et al., 2021; Schreiber-Gregory, 2018). According to Hair et al. (2021), the evaluation criteria are: VIF < 3 indicates no multicollinearity problem; 3 ≤ VIF < 5 indicates no severe multicollinearity; VIF ≥ 5 indicates a possible severe multicollinearity problem. As shown in Table 9, all VIF values in this study are significantly below the critical value of 3 (ranging from 1.238 to 1.914), indicating that there are no multicollinearity issues between the predictor variables.

**Table 9:** Assessment of structural model.

Collinearity (Inner VIF)	Construct	BPN	AM	LE	Criteria
	PAS	1.238	1.350	1.378	VIF < 3.0 (Hair et al., 2021)
	TAS	1.244	1.308	1.589	
	PS	1.248	1.572	1.573	
	BPN		1.805	1.914	
	AM			1.709	

R^2^	Construct	R^2^	R^2^ Adjusted	Criteria
	BPN	0.446	0.442	R^2^ ≈ 0.67:Strong R^2^ ≈ 0.33: Moderate R^2^ ≈ 0.19:Weak (Chin, 1998)
	AM	0.415	0.442	
	LE	0.410	0.403	

Effect size (f^2^)	Construct	BPN	AM	LE	Criteria
	PAS	0.260	0.021	0.002	f^2^ ≈ 0.35:Strong f^2^ ≈ 0.15: Moderate f^2^ ≈ 0.02:Weak (Chin, 1998)
	TAS	0.051	0.215	0.025	
	PS	0.090	0.000	0.013	
	BPN		0.060	0.023	
	AM			0.217	

Predictive relevance (Q^2^)	Construct	SS0	SSE	Q^2^(=1-SSE/SSO)	Criteria
	BPN	1248.000	865.989	0.306	Q^2^ >0 indicates predictive relevance (Hair et al., 2021)
	AM	1664.000	1234.863	0.258	
	LE	1248.000	848.497	0.320	

PAS, parental autonomy support; TAS, teacher autonomy support; PS, peer support; BPN, basic psychological needs; AM, autonomous motivation; AE, academic engagement.

The second step is to evaluate the significance and relevance of the path coefficients. The significance of the path coefficients is typically tested using bootstrapping (Chin, 1998), which generates samples by repeated resampling and calculates the standard errors of the path coefficients to obtain t-values and *p*-values. In this study, 5,000 bootstrap samples were used, and when the t-value exceeds the critical value of 1.96, the path coefficient is considered statistically significant.

**Figure 1:**
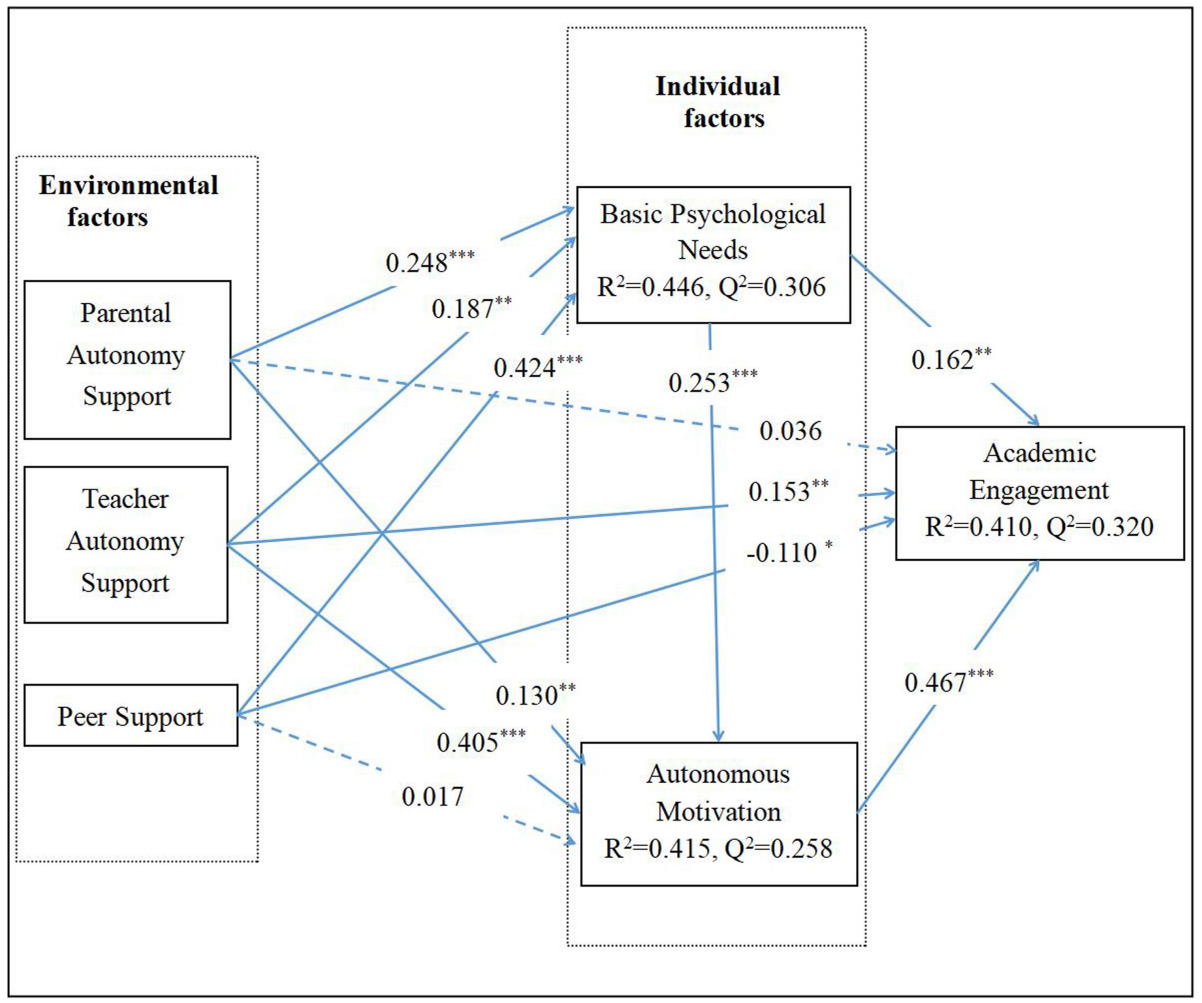
Inner model result of PLS-SEM. ***p < 0.001, **p < 0.01, *p < 0.05.

As shown in Table 10 and Figure 1, teacher autonomy support significantly positively predicts academic engagement (H1b: *β* = 0.153, *t* = 3.023, *p* < 0.01), peer support significantly negatively predicts academic engagement (H1c: *β* = −0.110, *t* = 2.269, *p* < 0.05), and parental autonomy support does not significantly predict academic engagement (H1a: *β* = 0.036, *t* = 0.692, *p* = 0.489). Therefore, H1b is supported, while H1a and H1c are not supported.

**Table 10:** Direct relationship.

Hypotheses	Relationship	*β*	SD	*t*	*p*-value	Decision
H1a	Parental autonomy support → Academic engagement	0.036	0.052	0.692	0.487	Not Supported
H1b	Teacher autonomy support → Academic engagement	0.153	0.051	3.023	0.003	Supported
H1c	Peer support → Learning engagement	−0.110	0.048	2.269	0.023	Not Supported

The third step is to assess the model’s explanatory power for endogenous variables using the coefficient of determination (R^2^). The R^2^ value indicates the degree to which the exogenous variables explain the endogenous variables and is an important indicator of model explanatory power. According to Chin (1998), the evaluation criteria for R^2^ values are 0.67, 0.33, and 0.19, representing strong, moderate, and weak explanatory power, respectively. As shown in Table 9, the R^2^ values for basic psychological needs (R^2^ = 0.446), autonomous motivation (R^2^ = 0.415), and academic engagement (R^2^ = 0.410) all demonstrate moderate explanatory power, indicating that the model has good explanatory power for the endogenous variables.

The fourth step is to evaluate the effect size of exogenous variables on endogenous variables using f^2^. f^2^ measures the extent of change in the R^2^ of the endogenous variable when an exogenous variable is included, while holding other predictors constant. According to Chin (1998), f^2^ values of 0.02, 0.15, and 0.35 represent small, medium, and large effect sizes, respectively. As shown in Table 9, parental autonomy support (f^2^ = 0.002) has a very weak effect on academic engagement, while teacher autonomy support (f^2^ = 0.025) and peer support (f^2^ = 0.013) have small effects on academic engagement. These results suggest that there may be more important mediators in the relationship between parental autonomy support, teacher autonomy support, peer support, and academic engagement.

The fifth step is to assess the model’s predictive ability for endogenous variables using predictive relevance (Q^2^). The Q^2^ value is calculated using the blindfolding procedure and measures the model’s predictive accuracy for the observed data (Chin, 1998). According to Hair et al. (2021), a Q^2^ value greater than 0 indicates strong predictive ability, meaning the model can effectively explain the variance of endogenous variables. As shown in Table 9, the Q^2^ values for basic psychological needs (Q^2^ = 0.306), autonomous motivation (Q^2^ = 0.258), and academic engagement (Q^2^ = 0.320) are all significantly greater than 0, indicating that the model has good predictive relevance for the endogenous variables.

Finally, the overall model fit is assessed through the Standardized Root Mean Square Residual (SRMR), Goodness of Fit (GOF), and Normed Fit Index (NFI) (Hu and Bentler, 1999). According to Henseler et al. (2016), an SRMR value < 0.08 indicates good model fit, while an SRMR value ≥ 0.08 suggests that the model needs further optimization. In this study, the SRMR value = 0.055, which is significantly lower than the threshold (0.08), indicating that the model fits well.

The GOF value is calculated by taking the geometric mean of the Average Variance Extracted (AVE) and the R^2^ values of all endogenous variables (Wetzels et al., 2009). That is, GOF = $\sqrt{\bar{AVE}\times \bar{R2}}$ . According to the evaluation criteria proposed by Wetzels et al. (2009), GOF values of 0.1, 0.25, and 0.36 indicate small, moderate, and strong fit, respectively. In this study, the calculated GOF value = 0.556, indicating an excellent overall model fit.

The NFI, as a relative fit index, is considered acceptable when NFI ≥ 0.8, according to Bentler (1990). The NFI value in this study is 0.843, further supporting the model’s validity.

Based on the results of the SRMR, GOF, and NFI, the model proposed in this study shows a good fit and confirms the robustness of the model structure.

### 4.6 Evaluation of mediating effect

After ensuring that the measurement model and structural model meet all quality criteria, the next step is to conduct the mediating effect analysis. This study used Smart-PLS 4.0 software and the bootstrapping technique for the mediating effect analysis, with 5,000 sampling iterations (Hair et al., 2021). The significance of the mediating effect is determined by the criterion that if the 95% confidence interval of the indirect effect does not include 0, the mediating effect is considered significant.

As shown in Table 11 and Figure 1, basic psychological needs mediate the relationship between parental autonomy support (H2a: *β* = 0.040, *t* = 2.670, *p* < 0.01, 95% CI = [0.013, 0.074]), teacher autonomy support (H2b: *β* = 0.030, *t* = 2.497, *p* < 0.05, 95% CI = [0.010, 0.057]), and peer support (H2c: *β* = 0.069, *t* = 2.876, *p* < 0.05, 95% CI = [0.024, 0.119]) with academic engagement. The t-values and *p*-values for these three paths are all significant, and the 95% confidence intervals do not include 0. Therefore, H2a, H2b, and H2c are supported.

**Table 11:** Mediation analysis.

Hypotheses	Relationship	*β*	SD	*t*	*p*-value	95%CI		Decision
						LLCI	ULCI	
H2a	PAS → BPN → AE	0.040	0.015	2.670	0.009	0.013	0.074	Supported
H2b	TAS → BPN → AE	0.030	0.012	2.497	0.013	0.010	0.057	Supported
H2c	PS → BPN → AE	0.069	0.024	2.876	0.004	0.024	0.119	Supported
H3a	PAS → AM → AE	0.061	0.023	2.691	0.007	0.016	0.105	Supported
H3b	TAS → AM → AE	0.189	0.034	5.646	0.000	0.130	0.259	Supported
H3c	PS → AM → AE	0.008	0.026	0.311	0.755	−0.040	0.059	Not Supported
H4a	PAS → BPN → AM → AE	0.029	0.008	3.468	0.001	0.014	0.048	Supported
H4b	TAS → BPN → AM → AE	0.022	0.003	3.097	0.002	0.010	0.038	Supported
H4c	PS → BPN → AM → AE	0.050	0.014	3.666	0.000	0.026	0.080	Supported

PAS, parental autonomy support; TAS, teacher autonomy support; PS, peer support; BPN, basic psychological needs; AM, autonomous motivation; AE, academic engagement.

Autonomous motivation mediates the relationship between parental autonomy support (H3a: *β* = 0.061, *t* = 2.691, *p* < 0.01, 95% CI = [0.016, 0.105]), teacher autonomy support (H3b: *β* = 0.189, *t* = 5.646, *p* < 0.001, 95% CI = [0.130, 0.259]), and academic engagement. The t-values and p-values for these two paths are significant, and the 95% confidence intervals do not include 0. Therefore, H3a and H3b are supported. However, the mediating effect of autonomous motivation in the relationship between peer support and academic engagement is not significant (H3c: *β* = 0.008, *t* = 0.311, *p* = 0.755, 95% CI = [−0.040, 0.059]), as the t-value and p-value are not significant, and the 95% confidence interval includes 0. Therefore, H3c is not supported.

In the chain mediation analysis, as shown in Table 11 and Figure 1, basic psychological needs and autonomous motivation mediate the relationship between parental autonomy support (H4a: *β* = 0.029, *t* = 3.468, *p* < 0.01, 95% CI = [0.001, 0.048]), teacher autonomy support (H4b: *β* = 0.022, *t* = 3.097, *p* < 0.05, 95% CI = [0.010, 0.038]), and peer support (H4c: *β* = 0.050, *t* = 3.666, *p* < 0.05, 95% CI = [0.026, 0.080]) with academic engagement. The t-values and p-values for these three chain mediation paths are all significant, and the 95% confidence intervals do not include 0. Therefore, H4a, H4b, and H4c are supported.

## 5 Discussion and conclusion

### 5.1 Parental autonomy support, teacher autonomy support, peer support, and academic engagement

This study shows that teacher autonomy support is significantly positively associated with academic engagement, while peer support is significantly negatively associated with academic engagement. Parental autonomy support is not significantly associated with academic engagement. Therefore, H1b is supported, while H1a and H1c are not supported.

#### 5.1.1 Teacher autonomy support and academic engagement

Research shows that, at the university level, teacher autonomy support is more effective in promoting academic engagement compared to parental and peer support, which is consistent with the findings of Guo et al. (2023), Parker et al. (2021), and Tao et al. (2022). The study by Shin and Chang (2022) also found that, compared to peer support, teacher autonomy support has a greater impact on academic engagement. Teachers are the most directly and specifically involved in students’ learning activities and processes (Wang et al., 2022). Teachers can directly influence student learning and promote engagement through their teaching style and teaching quality. Timely feedback and personalized guidance from teachers (Carvalho et al., 2021), can help students discover their strengths and weaknesses motivate their willingness to learn, and provide specific and targeted feedback to help students clarify learning goals, adjust learning strategies, and enhance engagement. Good teacher-student relationship (Quin, 2017) can also strengthen students’ sense of belonging in the classroom, thereby increasing engagement. Therefore, teachers, as the primary organizers, guides, and supervisors of student learning, play a significant role in providing diverse support that is associated with the promotion of academic engagement.

#### 5.1.2 Peer support and academic engagement

This study found that peer support is significantly negatively associated with academic engagement. There are several potential reasons for this.

First, in China’s unique “Gaokao” (college entrance exam) education system, there has always been a competitive relationship between peers, especially regarding academic performance (Zhao et al., 2015). The evaluation system in universities still places excessive emphasis on “individual competition,” and society as a whole exhibits a general state of “involution” (Yi et al., 2022), which leads to peer pressure that is associated with hindering academic engagement.

Second, the participants in this study are university students from western China, with 57.7% of the students coming from rural areas. Given the intense competition of the Gaokao, students from rural areas, during high school, focus almost exclusively on studying—there are few recreational activities beyond academics (Wang J., 2023). In the context of China’s unique educational background, most families view the Gaokao as a pivotal moment in life, especially for rural families, where it serves as an important stepping stone for social mobility (Liu and Helwig, 2022). These students rarely have free time during their 3 years of high school and even less time to engage in activities with their friends outside of studying (Han, 2024). Therefore, once they enter university, they may show a strong desire for freedom, along with a “rebellious” tendency for socializing and entertainment (Du F. et al., 2023). This stark contrast with their previous life can lead to feelings of guilt and self-blame, which makes peer support negatively associated with academic engagement.

Third, the study found that maintaining peer relationships requires significant time and energy, leading to an imbalance in time management. Students often struggle to balance academics and social activities, making it difficult to fully dedicate time and energy to their studies (Zhou et al., 2023), resulting in a decrease in academic engagement. Research has also shown that different peer groups have varying effects on students’ academic engagement. If the peer group does not prioritize academics and is more focused on social activities and various forms of entertainment, such as playing online games (Alam et al., 2023), such peer relationships are likely to lead to a decline in academic engagement.

Finally, the negative impact of peer support on academic engagement also reflects the growing independence of students over time. Every individual has limited time and energy, and it is necessary to balance life and study in order to realize one’s full potential.

#### 5.1.3 Parental autonomy support and academic engagement

The relationship between parental autonomy support and academic engagement is not significantly associated with academic engagement, and there are several potential reasons for this.

In China’s unique “Gaokao” (college entrance exam) education system, parents place a high value on their children’s education during the foundational stages, particularly focusing on their academic performance and learning status in middle and high school. (Chen et al., 2025a), in a study on Chinese high school students, found that parental autonomy support was significantly positively associated with academic engagement. However, once children enter university, it seems that parents’ expectations regarding their children’s academic achievements have been fulfilled, and the children no longer feel the pressure and guilt related to studying from their parents. Therefore, in the university stage, the relationship between parental autonomy support and academic engagement is no longer as strong.

Moreover, after entering university, students begin to live more independently, and their life and studies are gradually under their own control, leading to a decrease in direct parental influence. University students face more specialized and complex academic tasks, and most parents are no longer able to assist their children with their studies.

Although the direct influence of parental autonomy support on academic engagement has weakened, its impact typically occurs through indirect pathways. This is because parents have already subtly shaped their children’s study habits, attitudes, and values throughout their lives. Therefore, the influence of parents on their children is more evident through mediating effects, affecting their children’s attitudes and behaviors indirectly.

### 5.2 The mediating role of basic psychological needs

This study found that basic psychological needs mediate the relationship between parental autonomy support, teacher autonomy support, peer support, and academic engagement, therefore H2a, H2b, and H2c are supported. This finding is consistent with Self-Determination Theory (Ryan and Deci, 2017), which suggests that environments that support autonomy help fulfill basic psychological needs, thus promoting positive development in individuals. In this study, it was found that peer support has the greatest impact on basic psychological needs, followed by parental autonomy support, and lastly teacher autonomy support. This suggests that in equal relationships, fulfilling basic psychological needs (autonomy needs, competence needs, relatedness needs) is more conducive to personal growth.

Peer support in university provides students with a platform to share ideas, express emotions, and receive feedback, which not only enhances their emotional connection but also boosts their self-confidence and independence, providing strong supportive conditions for fulfilling basic psychological needs (Primana and Anisa, 2020). Compared to peers, both parents and teachers hold some level of authority, but parents’ recognition of their children’s emotional experiences, their reasonable explanation of rules and expectations, and their provision of opportunities for choice and autonomy still contribute to the fulfillment of basic psychological needs, thereby promoting academic engagement (Lan, 2023). The stronger the perception of teacher autonomy support, the more likely students are to emotionally identify with it, which further promotes the fulfillment of basic psychological needs (López-García et al., 2022), making them more likely to actively engage in various learning activities.

### 5.3 The mediating role of autonomous motivation

This study found that autonomous motivation mediates the relationship between parental autonomy support, teacher autonomy support, and academic engagement. However, autonomous motivation does not significantly mediate the relationship between peer support and academic engagement, so H3a and H3b are supported, while H3c is not supported.

Compared to parental autonomy support, teacher autonomy support has the greatest impact on students’ autonomous motivation, which indicates that the teacher-student learning relationship is the most direct and close. The primary role of teachers in the classroom is to help students build a knowledge system, understand and master knowledge, provide timely feedback, help students set learning goals, and clarify learning tasks. Teacher autonomy support plays an important role in stimulating students’ intrinsic motivation. Research by Yang and Du (2023) found that teacher autonomy support can influence students’ academic engagement through the mediating role of autonomous motivation, while the mediating effect of controlled motivation is not significant. Yang and Du argued that learners with high autonomous motivation, when they perceive teacher support in the learning environment, view this support as beneficial to their personal growth, which encourages them to adopt more effective learning strategies and time management, thereby allowing them to focus more on learning tasks.

Parents subtly influence their children’s learning autonomous motivation. Although most university students are away from home, parental autonomy support still affects their children’s autonomous motivation and thus promotes academic engagement. However, peer support does not significantly affect academic autonomous motivation, indicating that peers do not have a direct facilitating role in university students’ learning activities.

### 5.4 The chain mediating role of basic psychological needs and autonomous motivation

This study shows that both basic psychological needs and autonomous motivation play a chain mediating role in the relationship between parental autonomy support, teacher autonomy support, peer support, and academic engagement. Therefore, H4a, H4b, and H4c are supported. In other words, parental autonomy support, teacher autonomy support, and peer support can all promote the fulfillment of students’ basic psychological needs, which in turn fosters students’ motivation, thereby increasing their level of academic engagement. This finding is consistent with previous research (Chen et al., 2023; Ryan and Deci, 2017).

Self-Determination Theory posits that supportive external environments promote the satisfaction of basic psychological needs, which in turn facilitates the development of autonomous motivation, and autonomous motivation further influences individuals’ cognition and behavior (Deci et al., 1991; Ryan and Deci, 2017; Vansteenkiste et al., 2020). Research by Luo et al. (2014) found that teacher autonomy support perceived by students can alleviate academic burnout and improve academic performance through the chain mediation of basic psychological needs and autonomous motivation.

This highlights the importance of a supportive environment in fostering basic psychological needs and autonomous motivation. It also underscores that both basic psychological needs and autonomous motivation are essential for individual development. Together, they contribute to positive growth and help individuals realize their potential. Even if learning is a long-term and tedious process, it is possible to persist and achieve academic success.

## 6 Conclusion

(1) Teacher autonomy support is significantly positively associated with student engagement, while peer support is significantly negatively associated with academic engagement. Parental autonomy support is not significantly associated with academic engagement. (2) Basic psychological needs are associated with mediating the relationship between teacher autonomy support, peer support, and academic engagement. (3) Autonomous motivation is associated with mediating the relationship between parental autonomy support, teacher autonomy support, and student engagement. However, autonomous motivation is not significantly associated with mediating the relationship between peer support and academic engagement. (4) Basic psychological needs and autonomous motivation are associated with playing a chain mediating role in the relationship between parental autonomy support, teacher autonomy support, peer support, and academic engagement.

## 7 Implications

### 7.1 Theoretical implications

First, this study empirically examines the different roles that various levels of environmental factors (parents, teachers, peers) play in university students’ academic engagement, enriching and expanding the application of Ecological Systems Theory in the field of higher education. Second, this study verifies the chain mediating role of basic psychological needs and autonomous motivation in the relationship between autonomy-supportive environmental factors (parental autonomy support, teacher autonomy support, peer support) and individual positive development (academic engagement), deepening the application of Self-Determination Theory in a collectivist cultural context and further confirming the universality of Self-Determination Theory.

### 7.2 Practical implications

The results of this study indicate that university students’ academic engagement is influenced not only by external social support factors—such as parental autonomy support, teacher autonomy support, and peer support—but also relies on the satisfaction of their basic psychological needs and the stimulation of autonomous motivation. This highlights the importance of the synergistic interaction between external environmental factors and internal individual factors. Therefore, in educational practice, it is essential to foster a supportive environment. Through positive interactions with parents, teachers, and peers, university students can be guided to satisfy their psychological needs, enhance their autonomous motivation, and thereby achieve higher levels of academic engagement.

Firstly, in the context of family education, parents should shift their roles and become growth-minded individuals (Dweck, 2006). With the implementation of the “Double Reduction” policy in China’s basic education system (Yu et al., 2022), parents are expected to pay greater attention to their children’s learning and development during higher education, recognizing the crucial role that university education plays in their life planning. Parents should offer more respect and understanding, provide opportunities for autonomous choice, help their children identify their personal interests and strengths, and encourage them to continue cultivating expertise in their chosen academic fields. In this process, through emotional support, freedom of choice, and positive feedback, parental autonomy support facilitates the satisfaction of basic psychological needs and the development of autonomous motivation, enabling students to experience a sense of meaning and value through deep learning.

Secondly, in university teaching practices, teacher autonomy support has been shown to be a key factor in enhancing university students’ academic engagement. Teachers should continuously update their teaching philosophies and actively explore more flexible and diversified instructional approaches, fully leveraging the advantages of autonomy-supportive teaching to guide students toward active participation in the learning process. In recent years, service-learning—an instructional approach that integrates academic content with community service—has attracted increasing scholarly attention (Billig, 2000; Daum et al., 2022; Furco, 1996). Studies have shown that service-learning not only enhances students’ sense of competence, autonomy, and social responsibility, but also promotes their autonomous motivation and academic engagement (Hermoso and Secanell, 2024; Lobo-de-Diego et al., 2024). Ferrer et al. (2023) further emphasize that in service-learning programs, teachers provide timely emotional support and feedback, thereby fostering a student-centered learning environment that effectively stimulates students’ autonomous motivation, making them more willing to invest time and energy and achieve high-quality deep learning.

Finally, university students themselves should strengthen their self-awareness and growth mindset, actively explore their potential strengths and positive qualities, and learn to accept and appreciate themselves throughout the learning process. On this basis, they can develop a positive sense of self-identity. When individuals possess sound psychological quality and strong self-regulation skills, they are more likely to perceive and make use of autonomy support from parents, teachers, and peers. This enables them to internalize external supportive environments into intrinsic motivational forces, thereby continuously promoting their academic engagement and personal development.

## 8 Limitations and future research

First, this study uses a cross-sectional research design, which, to some extent, may not fully reflect the causal relationships between variables. Future research could adopt longitudinal designs or experimental studies to more accurately explore the causal relationships between variables. Second, the sample in this study is primarily from four universities in Guangxi, and therefore, the conclusions may not be fully generalizable to different regions or types of universities. Future research could expand the sample size to enhance the generalizability of the findings. Third, this study found that peer support is significantly negatively associated with academic engagement, and this result may be related to specific cultural or academic contexts. Future research could further explore the differences in the effects of peer support in various contexts. Additionally, the peer support scale used in this study primarily focuses on the emotional support dimension, which may not fully capture the multi-dimensional nature of peer support. Future research should consider using a more comprehensive peer support scale, including dimensions such as emotional support, academic support, informational support, and instrumental support, to more comprehensively assess the relationship between peer support and academic engagement. Finally, this study only examines the effects of parental autonomy support, teacher autonomy support, and peer support on academic engagement, without fully considering the interactions between these support factors. Future research should focus on analyzing the relationships between these support factors to gain a more comprehensive understanding of how multi-level environmental factors and individual factors are jointly associated with academic engagement.

## Data Availability

The raw data supporting the conclusions of this article will be made available by the authors, without undue reservation.

## References

[ref1] Abreu Alves S. Sinval J. Lucas Neto L. Marôco J. Gonçalves Ferreira A. Oliveira P. (2022). Burnout and dropout intention in medical students: the protective role of academic engagement. BMC Med. Educ. 22:83. doi: 10.1186/s12909-021-03094-9, PMID: 35130892 PMC8821797

[ref2] Adam M. S. Abd Hamid J. Khatibi A. Azam S. F. (2023). Autonomous motivation in blended learning: effects of teaching presence and basic psychological need satisfaction. Learn. Motiv. 83:101908. doi: 10.1016/j.lmot.2023.101908

[ref3] Alam M. I. Malone L. Nadolny L. Brown M. Cervato C. (2023). Investigating the impact of a gamified learning analytics dashboard: student experiences and academic achievement. J. Comput. Assist. Learn. 39, 1436–1449. doi: 10.1111/jcal.12853

[ref4] Bagozzi R. P. Yi Y. (1988). On the evaluation of structural equation models. J. Acad. Mark. Sci. 16, 74–94. doi: 10.1007/BF02723327

[ref5] Bai X. Gu X. (2024). Contribution of self-determining theory to K–12 students’ online learning engagements: research on the relationship among teacher support dimensions, students’ basic psychological needs satisfaction, and online learning engagements. Educ. Technol. Res. Dev. 72, 2939–2961. doi: 10.1007/s11423-024-10383-9, PMID: 40232369

[ref6] Bas G. Xu J. (2024). Interplay of teacher feedback, parental involvement and peer support on homework engagement of students. Br. Educ. Res. J. 50, 2735–2752. doi: 10.1002/berj.4049

[ref7] Benito-Gomez M. Lee G. Y. McCurdy A. L. Fletcher A. C. (2022). “If I hadn’t had that support system, I think I would have dropped out by now”: parental support in college and its implications for student adjustment. J. Fam. Issues 43, 3373–3394. doi: 10.1177/0192513X211044490

[ref8] Bentler P. M. (1990). Comparative fit indexes in structural models. Psychol. Bull. 107, 238–246. doi: 10.1037/0033-2909.107.2.238, PMID: 2320703

[ref9] Billig S. (2000). Research on K-12 school-based service-learning: the evidence builds. Phi Delta Kappan 658, 658–664.

[ref10] Brauer J. R. (2017). Cultivating conformists or raising rebels? Connecting parental control and autonomy support to adolescent delinquency. J. Res. Adolesc. 27, 452–470. doi: 10.1111/jora.12283, PMID: 28876516

[ref11] Brislin R. W. (1970). Back-translation for cross-cultural research. J. Cross-Cult. Psychol. 1, 185–216. doi: 10.1177/135910457000100301

[ref12] Bronfenbrenner U. (1979). The ecology of human development: Experiments by nature and design. Harvard: Harvard University Press.

[ref13] Carvalho C. Santos N. N. António R. Martins D. S. M. (2021). Supporting students’ engagement with teachers’ feedback: the role of students’ school identification. Educ. Psychol. 41, 863–882. doi: 10.1080/01443410.2020.1849564

[ref14] Chen C. Bian F. Zhu Y. (2023). The relationship between social support and academic engagement among university students: the chain mediating effects of life satisfaction and academic motivation. BMC Public Health 23:2368. doi: 10.1186/s12889-023-17301-3, PMID: 38031093 PMC10688496

[ref15] Chen T. Ding W. Yang Q. Chen Y. Li W. Xie R. (2025a). Longitudinal reciprocal relations between general basic psychological need satisfaction, social support, and academic engagement among Chinese adolescents. Soc. Psychol. Educ. 28:12. doi: 10.1007/s11218-024-09978-0

[ref16] Chen T. Xie R. Chen Y. Wenren S. Li W. Ding W. (2025b). The bidirectional relations between parental autonomy support, gratitude and academic engagement in Chinese adolescents. J. Youth Adolesc. 16:127. doi: 10.1007/s10964-024-02127-y, PMID: 39888574

[ref17] Chin W. W. (1998). “The partial least squares approach to structural equation modeling” in Modern methods for business research. ed. Marcoulides G. A. (Mahwah, NJ: Lawrence Erlbaum Associates).

[ref18] Chin W. W. (2009). “How to write up and report PLS analyses” in Handbook of partial least squares: Concepts, methods and applications. eds. Vinzi V. E. Chin W. W. Henseler J. Wang H. (Cham: Springer), 655–690.

[ref19] Daum D. N. Marttinen R. Banville D. (2022). Service-learning experiences for pre-service teachers: cultural competency and behavior management challenges when working with a diverse low-income community. Phys. Educ. Sport Pedagog. 27, 396–408. doi: 10.1080/17408989.2021.1891210

[ref20] Deci E. L. Ryan R. M. (1985). Intrinsic motivation and self-determination in human behavior. Perspect. Soc. Psychol. doi: 10.1007/978-1-4899-2271-7

[ref21] Deci E. L. Ryan R. M. (2000). The" what" and" why" of goal pursuits: human needs and the self-determination of behavior. Psychol. Inq. 11, 227–268. doi: 10.1207/S15327965PLI1104_01

[ref22] Deci E. L. Vallerand R. J. Pelletier L. G. Ryan R. M. (1991). Motivation and education: the self-determination perspective. Educ. Psychol. 26, 325–346. doi: 10.1080/00461520.1991.9653137

[ref23] Dong P. Pan Z. Yang Y. (2024). The protective roles of self-compassion and parental autonomy support against depressive symptoms in peer-victimized Chinese adolescent girls: a longitudinal study. J. Interpers. Violence 39, 3687–3711. doi: 10.1177/08862605241234344, PMID: 38444119

[ref24] Du W. Li Z. Xu Y. Chen C. (2023). The effect of parental autonomy support on grit: the mediating role of basic psychological needs and the moderating role of achievement motivation. Psychol. Res. Behav. Manag. 16, 939–948. doi: 10.2147/PRBM.S401667, PMID: 36992980 PMC10042245

[ref25] Du F. Wang W. Dong X. (2023). ““Happy” college students” in Chinese people’s time use and their quality of life: Research report of Chinese time use survey. eds. Du F. Wang W. Dong X. (Cham: Springer), 159–175.

[ref26] Dweck C. S. (2006). Mindset: The new psychology of success. New York: Random house.

[ref27] Espinoza-Gutiérrez R. Baños R. Calleja-Núñez J. J. Granero-Gallegos A. (2024). Effect of teaching style on academic self-concept in Mexican university students of physical education: multiple mediation of basic psychological needs and motivation. Espiral Cuadernos del Prof. 17, 46–61. doi: 10.25115/ecp.v17i36.10087

[ref28] Ferrer C. M. S. Romero M. P. A. Torres M. F. García D. S. (2023). Competencies, motivation and engagement of university students through service-learning experiences Competencias, motivación y compromiso con el trabajo de estudiantes universitarios a través de experiencias de Aprendizaje-Servicio. Espiral. Cuadernos del Prof. 16, 25–40. doi: 10.25115/ecp.v16i33.9192

[ref29] Fornell C. Larcker D. F. (1981). Evaluating structural equation models with unobservable variables and measurement error. J. Mark. Res. 18, 39–50. doi: 10.1177/002224378101800104

[ref30] Furco A. (1996). Service-learning: A balanced approach to experiential education. ETR Associates.

[ref31] Gagné M. (2003). The role of autonomy support and autonomy orientation in prosocial behavior engagement. Motiv. Emot. 27, 199–223. doi: 10.1023/A:1025007614869

[ref32] Guo Q. Samsudin S. Yang X. Gao J. Ramlan M. A. Abdullah B.. (2023). Relationship between perceived teacher support and student engagement in physical education: a systematic review. Sustain. For. 15:6039. doi: 10.3390/su15076039

[ref33] Guoxia W. Yang Z. (2024). A Meta-analysis on teacher autonomy support and student academic achievement: the mediating effect of psychological need satisfaction, academic motivation, and academic engagement. Front. Educ. China 19, 403–420. doi: 10.3868/s110-010-024-0022-3

[ref34] Hair J. F. Hult G. T. M. Ringle C. M. Sarstedt M. Danks N. P. Ray S. (2021). Partial least squares structural equation modeling (PLS-SEM) using R: a workbook. Available online at: https://library.oapen.org/handle/20.500.12657/51463 (Accessed September 5, 2024).

[ref35] Hair J. F. Sarstedt M. Hopkins L. Kuppelwieser V. G. (2014). Partial least squares structural equation modeling (PLS-SEM): an emerging tool in business research. Eur. Bus. Rev. 26, 106–121. doi: 10.1108/EBR-10-2013-0128

[ref9001] Hair J. F. Jr. Hult G. T. M. Ringle C. Sarstedt M. (2017). A primer on partial least squares structural equation modeling (PLS-SEM). Thousand Oaks, CA: Sage Publications.

[ref36] Han Z. (2024). Academic stress and coping strategies of Chinese high school students-a qualitative investigation. Adv. Educ. Hum. Soc. Sci. Res. 12:270. doi: 10.56028/aehssr.12.1.270.2024

[ref37] Harman H. H. (1976). Modern factor analysis. Chicago, IL: University of Chicago press.

[ref38] Henseler J. Hubona G. Ray P. A. (2016). Using PLS path modeling in new technology research: updated guidelines. Ind. Manag. Data Syst. 116, 2–20. doi: 10.1108/imds-09-2015-0382

[ref39] Henseler J. Ringle C. M. Sarstedt M. (2015). A new criterion for assessing discriminant validity in variance-based structural equation modeling. J. Acad. Mark. Sci. 43, 115–135. doi: 10.1007/s11747-014-0403-8

[ref40] Hermoso R. V. Secanell I. L. (2024). Learning-service in High-school: Systematic review from physical education [El aprendizaje-servicio en Educación Secundaria: Revisión sistemática desde la Educación Física]. Espiral. Cuadernos Prof. 17:9659. doi: 10.25115/ecp.v17i35.9659

[ref41] Hoferichter F. Kulakow S. Hufenbach M. C. (2021). Support from parents, peers, and teachers is differently associated with middle school students’ well-being. Front. Psychol. 12:758226. doi: 10.3389/fpsyg.2021.758226, PMID: 34925161 PMC8674184

[ref42] Hu L. T. Bentler P. M. (1999). Cutoff criteria for fit indexes in covariance structure analysis: conventional criteria versus new alternatives. Struct. Equ. Model. Multidiscip. J. 6, 1–55. doi: 10.1080/10705519909540118

[ref43] Jiang R. Fan R. Zhang Y. Li Y. (2022). Understanding the serial mediating effects of career adaptability and career decision-making self-efficacy between parental autonomy support and academic engagement in Chinese secondary vocational students. Front. Psychol. 13:953550. doi: 10.3389/fpsyg.2022.953550, PMID: 36033072 PMC9402251

[ref44] Kahu E. R. Nelson K. (2018). Student engagement in the educational interface: understanding the mechanisms of student success. High. Educ. Res. Dev. 37, 58–71. doi: 10.1080/07294360.2017.1344197

[ref45] Kotera Y. Conway E. Green P. (2023). Construction and factorial validation of a short version of the academic motivation scale. Br. J. Guid. Couns. 51, 274–283. doi: 10.1080/03069885.2021.1903387

[ref46] Krosnick J. A. (1991). Response strategies for coping with the cognitive demands of attitude measures in surveys. Appl. Cogn. Psychol. 5, 213–236. doi: 10.1002/acp.2350050305

[ref47] Kuh G. D. (2001). Assessing what really matters to student learning inside the national survey of student engagement. Change 33, 10–17. doi: 10.1080/00091380109601795

[ref48] Lan X. (2023). Does peer acceptance promote active academic engagement in early adolescence? A robust investigation based on three independent studies. Personal. Individ. Differ. 203:112012. doi: 10.1016/j.paid.2022.112012

[ref49] Lazarsfeld P. F. Berelson B. Gaudet H. (1968). The people’s choice: how the voter makes up his mind in a presidential campaign. New York, NY: Columbia University Press.

[ref50] Lee J. A. L. More S. J. Cotiw-an B. S. (1999). Problems translating a questionnaire in a cross-cultural setting. Prev. Vet. Med. 41, 187–194. doi: 10.1016/S0167-5877(99)00041-0, PMID: 10448945

[ref51] Little R. J. Rubin D. B. (2019). Statistical analysis with missing data. Hoboken, NJ: John Wiley and Sons.

[ref52] Liu G. X. Y. Helwig C. C. (2022). Autonomy, social inequality, and support in Chinese urban and rural adolescents’ reasoning about the Chinese college entrance examination (Gaokao). J. Adolesc. Res. 37, 639–671. doi: 10.1177/0743558420914082

[ref54] Lobo-de-Diego F. E. Monjas-Aguado R. Manrique-Arribas J. C. (2024). Experiences of service-learning in the initial training of physical education teachers [Experiencias de Aprendizaje-Servicio en la formación inicial del profesorado de Educación Física]. Espiral. Cuadernos Prof. 17:9688. doi: 10.25115/ecp.v17i35.9688

[ref55] López-García G. D. Carrasco-Poyatos M. Burgueño R. Granero-Gallegos A. (2022). Teaching style and academic engagement in pre-service teachers during the COVID-19 lockdown: mediation of motivational climate. Front. Psychol. 13:992665. doi: 10.3389/fpsyg.2022.992665, PMID: 36312149 PMC9614661

[ref56] Luo Y. Zhao M. Wang Z. H. (2014). Effect of perceived teacher autonomy support on academic burnout among junior high school students: the mediating roles of basic psychological needs, and autonomous motivation. Psychol. Dev. Educ. 30, 312–321. doi: 10.16187/j.cnki.issn1001-4918.2014.03.010

[ref57] Luruli K. Mostert K. Jacobs M. (2020). Testing a structural model for study demands and resources, study engagement and well-being of first-year university students. J. Psychol. Afr. 30, 179–186. doi: 10.1080/14330237.2020.1767925

[ref58] Mageau G. A. Ranger F. Joussemet M. Koestner R. Moreau E. Forest J. (2015). Validation of the perceived parental autonomy support scale (P-PASS). Canad. J. Behav. Sci. 47, 251–262. doi: 10.1037/a0039325

[ref59] Martínez-López Z. Moran V. E. Mayo M. E. Villar E. Tinajero C. (2024). Perceived social support and its relationship with self-regulated learning, goal orientation self-management, and academic achievement. Eur. J. Psychol. Educ. 39, 813–835. doi: 10.1007/s10212-023-00752-y

[ref60] Mayer A. (2021). Reducing respondents’ perceptions of bias in survey research. Methodol. Innov. 14:20597991211055952. doi: 10.1177/20597991211055952

[ref61] Meade A. W. Craig S. B. (2012). Identifying careless responses in survey data. Psychol. Methods 17, 437–455. doi: 10.1037/a0028085, PMID: 22506584

[ref62] Oppenheimer D. M. Meyvis T. Davidenko N. (2009). Instructional manipulation checks: detecting satisficing to increase statistical power. J. Exp. Soc. Psychol. 45, 867–872. doi: 10.1016/j.jesp.2009.03.009

[ref63] Pan X. Yao Y. (2023). Enhancing Chinese students’ academic engagement: the effect of teacher support and teacher–student rapport. Front. Psychol. 14:1188507. doi: 10.3389/fpsyg.2023.1188507, PMID: 37397305 PMC10311437

[ref64] Park D. Gunderson E. A. Maloney E. A. Tsukayama E. Beilock S. L. Duckworth A. L.. (2023). Parental intrusive homework support and math achievement: does the child’s mindset matter? Dev. Psychol. 59, 1249–1267. doi: 10.1037/dev0001522, PMID: 37166869 PMC10835763

[ref65] Parker J. S. Parris L. Lau M. Dobbins A. Shatz L. Porush S.. (2021). Perceived teacher autonomy support and self-determination skill expression: predictors of student engagement among African American high school students. J. Black Psychol. 47, 445–475. doi: 10.1177/00957984211009190

[ref66] Podsakoff P. M. Mac Kenzie S. B. Lee J.-Y. Podsakoff N. P. (2003). Common method biases in behavioral research: a critical review of the literature and recommended remedies. J. Appl. Psychol. 88, 879–903. doi: 10.1037/0021-9010.88.5.879, PMID: 14516251

[ref67] Primana L. Anisa A. (2020). The importance of basic psychological needs satisfaction of peer support for meaningful learning and college student engagement. Hauppauge, NY: Nova Science Publishers.

[ref68] Quin D. (2017). Longitudinal and contextual associations between teacher–student relationships and student engagement: a systematic review. Rev. Educ. Res. 87, 345–387. doi: 10.3102/0034654316669434

[ref69] Räisänen M. Postareff L. Lindblom-Ylänne S. (2021). Students’ experiences of study-related exhaustion, regulation of learning, peer learning and peer support during university studies. Eur. J. Psychol. Educ. 36, 1135–1157. doi: 10.1007/s10212-020-00512-2

[ref70] Ryan R. M. Deci E. L. (2000). Self-determination theory and the facilitation of intrinsic motivation, social development, and well-being. Am. Psychol. 55, 68–78. doi: 10.1037/0003-066X.55.1.68, PMID: 11392867

[ref71] Ryan R. M. Deci E. L. (2017). Self-determination theory: basic psychological needs in motivation, development, and wellness. New York, NY: Guilford Publications.

[ref72] Ryan R. M. Deci E. L. (2020). Intrinsic and extrinsic motivation from a self-determination theory perspective: definitions, theory, practices, and future directions. Contemp. Educ. Psychol. 61:101860. doi: 10.1016/j.cedpsych.2020.101860

[ref73] Sağkal A. S. Sönmez M. T. (2022). The effects of perceived parental math support on middle school students’ math engagement: the serial multiple mediation of math self-efficacy and math enjoyment. Eur. J. Psychol. Educ. 37, 341–354. doi: 10.1007/s10212-020-00518-w

[ref74] Sánchez-Martín M. Olmedo Moreno E. M. Gutiérrez-Sánchez M. Navarro-Mateu F. (2024). EQUATOR-network: a roadmap toimprove the quality and transparency of research reporting. Espiral. Cuadernos Prof. 17, 108–116. doi: 10.25115/ecp.v17i35.9529

[ref9002] Schaufeli W. B. Bakker A. B. Salanova M. (2006). The Measurement of Work Engagement With a Short Questionnaire: A Cross-National Study. Educational and Psychological Measurement. 66, 701–716. doi: 10.1177/0013164405282471

[ref75] Schaufeli W. B. Martinez I. M. Pinto A. M. Salanova M. Bakker A. B. (2002). Burnout and engagement in university students: a cross-national study. J. Cross-Cult. Psychol. 33, 464–481. doi: 10.1177/0022022102033005003

[ref76] Schreiber-Gregory D. N. (2018). Ridge regression and multicollinearity: an in-depth review. Model. Assist. Stat. Appl. 13, 359–365. doi: 10.3233/MAS-180446

[ref77] Schweder S. Raufelder D. (2022). Examining positive emotions, autonomy support and learning strategies: self-directed versus teacher-directed learning environments. Learn. Environ. Res. 25, 507–522. doi: 10.1007/s10984-021-09378-7

[ref78] Seligman M. E. Csikszentmihalyi M. (2000). Positive psychology: An introduction. Washington, DC: American Psychological Association. 10.1037//0003-066x.55.1.5 11392865

[ref79] Shin H. Chang Y. (2022). Relational support from teachers and peers matters: links with different profiles of relational support and academic engagement. J. Sch. Psychol. 92, 209–226. doi: 10.1016/j.jsp.2022.03.006, PMID: 35618371

[ref80] Siacor K. H. Ng B. (2024). Fostering student motivation and engagement through teacher autonomy support: a self-determination theory perspective. Int. J. Instr. 17, 583–598. doi: 10.29333/iji.2024.17232a

[ref81] Simon P. D. Salanga M. G. C. (2021). Validation of the five-item learning climate questionnaire as a measure of teacher autonomy support in the classroom. Psychol. Sch. 58, 1919–1931. doi: 10.1002/pits.22546

[ref82] Soenens B. Vansteenkiste M. (2005). Antecedents and outcomes of self-determination in 3 life domains: the role of parents' and teachers' autonomy support. J. Youth Adolesc. 34, 589–604. doi: 10.1007/s10964-005-8948-y

[ref83] Soenens B. Vansteenkiste M. Lens W. Luyckx K. Goossens L. Beyers W.. (2007). Conceptualizing parental autonomy support: adolescent perceptions of promotion of independence versus promotion of volitional functioning. Dev. Psychol. 43, 633–646. doi: 10.1037/0012-1649.43.3.633, PMID: 17484576

[ref84] Sueb R. Hashim H. Hashim K. S. Izam M. M. (2020). Excellent teachers' strategies in managing students' misbehaviour in the classroom. Asian J. Univ. Education 16:46. doi: 10.24191/ajue.v16i1.8982

[ref85] Tabiś K. Poprawa R. (2023). Satisfaction and frustration of basic psychological needs and the risk of relapse in alcohol dependence. Alcohol. Drug Addict. 36, 21–40. doi: 10.5114/ain.2023.130374

[ref86] Tao Y. Meng Y. Gao Z. Yang X. (2022). Perceived teacher support, student engagement, and academic achievement: a meta-analysis. Educ. Psychol. 42, 401–420. doi: 10.1080/01443410.2022.2033168, PMID: 40101104

[ref87] Trowler V. (2010). Student engagement literature review. High. Educ. Acad. 11, 1–15.

[ref88] Tunca A. (2024). Adolescents’ perceptions of autonomy-supportive parents and social-emotional health: serial multiple mediation of positive stress and mental toughness. J. Psychol. Couns. Sch. 34, 399–414. doi: 10.1177/20556365241295297

[ref89] Ulmanen S. Tikkanen L. Pyhältö K. (2024). Sense of relatedness and study engagement as mediators between students’ peer support and life satisfaction. Eur. J. Psychol. Educ. 39, 2603–2617. doi: 10.1007/s10212-024-00858-x, PMID: 40232369

[ref90] Vallerand R. J. Pelletier L. G. Blais M. R. Briere N. M. Senecal C. Vallieres E. F. (1992). The academic motivation scale: a measure of intrinsic, extrinsic, and amotivation in education. Educ. Psychol. Meas. 52, 1003–1017. doi: 10.1177/0013164492052004025

[ref91] Vansteenkiste M. Ryan R. M. Soenens B. (2020). Basic psychological need theory: advancements, critical themes, and future directions. Motiv. Emot. 44, 1–31. doi: 10.1007/s11031-019-09818-1

[ref92] Vargas-Madriz L. F. Konishi C. Wong T. K. (2024). A meta-analysis of the association between teacher support and school engagement. Soc. Dev. 33:e12745. doi: 10.1111/sode.12745

[ref93] Virtanen T. E. Lerkkanen M.-K. Poikkeus A.-M. Kuorelahti M. (2014). Student behavioral engagement as a mediator between teacher, family, and peer support and school truancy. Learn. Individ. Differ. 36, 201–206. doi: 10.1016/j.lindif.2014.09.001

[ref94] Von Elm E. Altman D. G. Egger M. Pocock S. J. Gøtzsche P. C. Vandenbrouckef J. P. (2008). The strengthening the reporting of observational studies in epidemiology (STROBE) statement: Guidelinesfor reporting observational studies. Bull. World Health Organ. 85, 867–872. doi: 10.2471/BLT.07.045120, PMID: 18038077 PMC2636253

[ref95] Wang J. (2023). Chinese parental academic socialization prior to college entrance examination: insights from urban and rural areas. J. Fam. Stud. 29, 389–406. doi: 10.1080/13229400.2021.1934516

[ref96] Wang Z. (2023). Achieving universal higher education in China: characteristics, challenges, and future directions. Pac. Int. J. 6, 125–129. doi: 10.55014/pij.v6i4.490

[ref97] Wang J. Zhang X. Zhang L. J. (2022). Effects of teacher engagement on students’ achievement in an online English as a foreign language classroom: the mediating role of autonomous motivation and positive emotions. Front. Psychol. 13:950652. doi: 10.3389/fpsyg.2022.950652, PMID: 35846620 PMC9284120

[ref98] Wei Z. F. (2023). Effect of self-control on academic procrastination in college students: the chain mediating role of mobile phone addiction and learning engagement. Chin. J. Clin. Psych. 31, 1248–1252. doi: 10.16128/j.cnki.1005-3611.2023.05.044

[ref99] Wentzel K. R. Battle A. Russell S. L. Looney L. B. (2010). Social supports from teachers and peers as predictors of academic and social motivation. Contemp. Educ. Psychol. 35, 193–202. doi: 10.1016/j.cedpsych.2010.03.002

[ref100] Wetzels M. Odekerken-Schröder G. Van Oppen C. (2009). Using PLS path modeling for assessing hierarchical construct models: guidelines and empirical illustration. MIS Q. 33:177. doi: 10.2307/20650284, PMID: 39964225

[ref101] Williams G. C. Deci E. L. (1996). Internalization of biopsychosocial values by medical students: a test of self-determination theory. J. Pers. Soc. Psychol. 70, 767–779. doi: 10.1037/0022-3514.70.4.767, PMID: 8636897

[ref102] Xie X. Guo J. (2023). Influence of teacher-and-peer support on positive academic emotions in EFL learning: the mediating role of mindfulness. Asia Pac. Educ. Res. 32, 439–447. doi: 10.1007/s40299-022-00665-2

[ref103] Xin Z. (2022). Perceived social support and college student engagement: moderating effects of a grateful disposition on the satisfaction of basic psychological needs as a mediator. BMC Psychol. 10:298. doi: 10.1186/s40359-022-01015-z, PMID: 36503628 PMC9743595

[ref104] Xu X. Wu Z. Wei D. (2024). Perceived teacher support and student engagement: the chain mediating effect of basic psychological needs satisfaction and learning drive. J. Psychol. Afr. 34, 73–79. doi: 10.1080/14330237.2024.2311984

[ref105] Yan J. Zhang T. Zhou X. Li H. (2025). Whose autonomy support is more effective in promoting exercise adherence in higher vocational college students-based on self-determined theory. BMC Public Health 25:395. doi: 10.1186/s12889-025-21587-w, PMID: 39885495 PMC11783744

[ref106] Yang D. Chen P. Wang K. Li Z. Zhang C. Huang R. (2023). Parental involvement and student engagement: a review of the literature. Sustain. For. 15:5859. doi: 10.3390/su15075859

[ref107] Yang Y. Du C. (2023). The predictive effect of perceived teacher support on college EFL learners’ online learning engagement: autonomous and controlled motivation as mediators. J. Multiling. Multicult. Dev. 1:879. doi: 10.1080/01434632.2023.2259879, PMID: 40101104

[ref108] Yang H. Xiang J. (2025). Peer support and academic engagement: the moderated mediation model for college students. Psychol. Sch. 62, 281–295. doi: 10.1002/pits.23324

[ref109] Yi D. Wu J. Zhang M. Zeng Q. Wang J. Liang J.. (2022). Does involution cause anxiety? An empirical study from Chinese universities. Int. J. Environ. Res. Public Health 19:9826. doi: 10.3390/ijerph19169826, PMID: 36011462 PMC9408648

[ref111] Yu S. He Z. Z. Cui T. He J. (2024). Basic psychological needs as an explanation of body image and eating pathways to adolescent adjustment: a prospective study testing an integrated self-determination theory model: first author. Motiv. Emot. 48, 170–185. doi: 10.1007/s11031-024-10063-4

[ref112] Yu C. F. Li X. Zhang W. (2015). Predicting adolescent problematic online game use from teacher autonomy support, basic psychological needs satisfaction, and school engagement: a 2-year longitudinal study. Cyberpsychol. Behav. Soc. Netw. 18, 228–233. doi: 10.1089/cyber.2014.0385, PMID: 25803769

[ref113] Yu S. Zheng J. Xu Z. Zhang T. (2022). The transformation of parents’ perception of education involution under the background of “double reduction” policy: the mediating role of education anxiety and perception of education equity. Front. Psychol. 13:800039. doi: 10.3389/fpsyg.2022.800039, PMID: 35664177 PMC9161288

[ref115] Zhang B. G. Qian X. F. (2022). Perceived teacher’s support and engagement among students with obesity in physical education: the mediating role of basic psychological needs and autonomous motivation. J. Sports Sci. 40, 1901–1911. doi: 10.1080/02640414.2022.2118935, PMID: 36062925

[ref116] Zhang R. Qiu Z. Li Y. Liu L. Zhi S. (2021). Teacher support, peer support, and externalizing problems among left-behind children in rural China: sequential mediation by self-esteem and self-control. Child Youth Serv. Rev. 121:105824. doi: 10.1016/j.childyouth.2020.105824

[ref117] Zhao X. Huang S. Shi C. (2024). The effect of parental and teacher autonomy support and core self-evaluations: a three-wave longitudinal study of middle students’ career adaptability. Front. Psychol. 15:1404478. doi: 10.3389/fpsyg.2024.1404478, PMID: 39403239 PMC11472576

[ref118] Zhao X. Selman R. L. Haste H. (2015). Academic stress in Chinese schools and a proposed preventive intervention program. Cogent Educ. 2:1000477. doi: 10.1080/2331186X.2014.1000477

[ref119] Zhou Y. Meng X. Wang J. Mo X. Jiang S. Dai C.. (2023). Daily peer relationships and academic achievement among college students: a social network analysis based on behavioral big data. Sustain. For. 15:15762. doi: 10.3390/su152215762

[ref120] Zimet G. D. Dahlem N. W. Zimet S. G. Farley G. K. (1988). The multidimensional scale of perceived social support. J. Pers. Assess. 52, 30–41. doi: 10.1207/s15327752jpa5201_2 2280326

